# Detecting Matching Blunders of Multi-Source Remote Sensing Images via Graph Theory

**DOI:** 10.3390/s20133712

**Published:** 2020-07-02

**Authors:** Cailong Deng, Xiuxiao Yuan, Lixia Deng, Jun Chen

**Affiliations:** School of Remote Sensing and Information Engineering, Wuhan University, Wuhan 430079, China; dcl@whu.edu.cn (C.D.); denglixia@whu.edu.cn (L.D.); jchen_cug@whu.edu.cn (J.C.)

**Keywords:** multi-source remote sensing images, matching blunder detection, complete graph, TIN graph, RANSAC

## Abstract

Large radiometric and geometric distortion in multi-source images leads to fewer matching points with high matching blunder ratios, and global geometric relationship models between multi-sensor images are inexplicit. Thus, traditional matching blunder detection methods cannot work effectively. To address this problem, we propose two matching blunder detection methods based on graph theory. The proposed methods can build statistically significant clusters in the case of few matching points with high matching blunder ratios, and use local geometric similarity constraints to detect matching blunders when the global geometric relationship is not explicit. The first method (named the complete graph-based method) uses clusters constructed by matched triangles in complete graphs to encode the local geometric similarity of images, and it can detect matching blunders effectively without considering the global geometric relationship. The second method uses the triangular irregular network (TIN) graph to approximate a complete graph to reduce to computational complexity of the first method. We name this the TIN graph-based method. Experiments show that the two graph-based methods outperform the classical random sample consensus (RANSAC)-based method in recognition rate, false rate, number of remaining matching point pairs, dispersion, positional accuracy in simulated and real data (image pairs from Gaofen1, near infrared ray of Gaofen1, Gaofen2, panchromatic Landsat, Ziyuan3, Jilin1and unmanned aerial vehicle). Notably, in most cases, the mean false rates of RANSAC, the complete graph-based method and the TIN graph-based method in simulated data experiments are 0.50, 0.26 and 0.14, respectively. In addition, the mean positional accuracy (RMSE measured in units of pixels) of the three methods is 2.6, 1.4 and 1.5 in real data experiments, respectively. Furthermore, when matching blunder ratio is no higher than 50%, the computation time of the TIN graph-based method is nearly equal to that of the RANSAC-based method, and roughly 2 to 40 times less than that of the complete graph-based method.

## 1. Introduction

Obtaining stable matching points between images is crucial for the accuracy and efficiency of current applications of advanced photogrammetric and computer vision methods. However, due to the large time span, big difference in shooting angles, and different platforms and sensors, there is large radiometric and geometric distortion among multi-source images. Matching blunders are unavoidable, and the matching blunders presented in multi-sensor images have two distinct features. Firstly, there are fewer matching points with higher matching blunder ratios. Matching blunder ratio represents the ratio of number of matching blunders to the number of whole matching point pairs. Secondly, it is difficult to derive the transformation models between images from different sensors, that is, the global geometric relationship models for matching blunder detection in multi-source images cannot be obtained easily, and sometimes even do not exist. The geometric relationship model is a mathematical model under which two corresponding matching points are precisely related. Various researchers have made contributions to solving the matching blunder detection problems of multi-sensor images, and their research can be summarized as follows.

(1) For high matching blunder ratios, random sample consensus (RANSAC) [1] is a powerful tool since it is not sensitive to matching blunder ratios. If all the inliers have a precise geometric relationship model between images, then RANSAC can always distinguish outliers from inliers. Many researchers modified either the sampling process [2,3,4] or the geometric relationship model [5,6,7,8,9] of RANSAC to make it suitable for certain applications. However, RANSAC and its variants cannot accurately describe local deformation in images, and are likely to fall into the local minimum when there are more than two models of a comparable number of observations. Other than RANSAC and its variants, there are other matching blunder detection methods based on global approximate models. Vector field consensus (VFC) [10] was robust when used with a large number of outliers, but only experimented in close-range images. Based on the *l_q_*-estimator and an affine model, Li et al. [11] proposed a robust feature-matching method which can deal with up to 90% outliers, while the experimental image pairs were from the same sensor. It is difficult for the above-mentioned methods based on global approximate models to deal with large local geometric deformation caused by large elevation differences. The global geometric relationship models for matching blunder detection in particular cannot be obtained easily in multi-source images, or even do not exist.

(2) As there is local deformation in images and sometimes no global geometric relationship models for detecting matching blunders, many researchers have derived local similarity-based methods. These methods are generally realized via the neighborhood relations of matching points in the local image. For example, Zhang et al. [12,13] eliminated mismatching points quickly based on the local vector field, while mismatching points were assumed to be normally distributed. Chen et al. [14] alternately carried out the Hough transform and inverted it to establish local feature correspondences, as well as improved matching precision and recall rate. However, local similarity-based methods generally depend on the statistical properties of matching blunders. If there are insufficient matching points, matching blunders will be concealed and cannot be detected.

Graph-based methods [15] pave a possible way to solve this problem as they can build plenty of redundancy of measurements to calculate meaningful statistical parameters, which are very helpful for matching blunder detection. Furthermore, the redundancy of measurements for local geometric similarity can be built via the relation information (such as distance or angle) associated with the edges in the graphs [16]. Some graph-based methods have found feature correspondences based on the statistical clusters of edge-to-edge (or pairwise) similarities between matching points. For example, spectral graph matching (SGM) [17], balanced graph matching (BGM) [18], probabilistic graph matching (PGM) [19], reweighted random walks for graph matching (RRWM) [20], graph transformation matching (GTM) [21], and max-pooling matching (MPM) [22] considered pairwise similarities, such as the distance of an edge pair. However, the methods based on pairwise relationships are only rotation-invariant, and neither scale-invariant nor affine-invariant. Other graph-based methods have been studied to solve the limitation, by considering affine-invariant similarities between tuples of feature points. For instance, weighted graph transformation matching (WGTM) [23], efficient high order matching (EHOM) [24], high-order graph matching (HOGM) [16], and reweighted random walks hyper-graph matching (RRWHM) [25] considered angle similarities of triangles formed by triple nodes between two graphs. These methods have shown good performance in accuracy. However, they have barely been experimented in multi-source remote sensing images, and generally have high computational complexity, especially when the matching blunder ratio is high. In addition, Chen et al. [26] used an affinity tensor to represent the complete graph for detecting matching blunders in multi-sensor images, while their tenor power iteration method is very time consuming.

To deal with the above distinct features of matching blunders in multi-sensor images, i.e., fewer matching points with higher matching blunder ratios and unknown global geometric relationship models, we study a complete graph-based method which adopts matched triangles between two complete graphs to encode the local geometric similarity of images to handle unknown global geometric relationship models. Though there are fewer matching points, statistically significant clusters of matched triangles can be constructed by the graphs to provide redundant measurements for matching blunder detection. Meanwhile, the clusters of matched triangles can also identify matching blunders by node attributes of the induced graph in spite of high matching blunder ratios. The complete graph-based method can obtain robust experimental results of matching blunder detection in multi-source images.

Besides, in the case of relatively low matching blunder ratios, a triangular irregular network (TIN) graph can effectively retain geometric properties of a complete graph and hugely reduce computational complexity of encoding the local similarity of an image. Thus, we propose the TIN graph-based method, using matched triangles between two TIN graphs for matching blunder detection. The TIN graph-based method can achieve a good balance between robustness and efficiency in the experiments of multi-source images. According to different graph forms adopted in graph theory, there are two graph theory-based methods in this paper: the complete graph-based method for matching blunder detection (COM graph), and the upgraded version of COM graph, the TIN graph-based method, which is also used for matching blunder detection (TIN graph). On the whole, the novelties of the proposed methods are that they can build statistically significant clusters of matched triangles in the case of few matching points with high matching blunder ratios, and use local geometric similarity constraints to detect matching blunders when the global geometric relationship is not explicit. In most cases, the proposed methods have nearly equal recognition rates to RANSAC, but can improve the performance of detecting matching blunders in a false rate and positional accuracy by about two times that of RANSAC.

This paper is organized as follows: Firstly, the workflow and principle of TIN graph are described in detail, and the differences between TIN graph and COM graph are analyzed (Section 2). Secondly, extensive experiments of simulated data and real data are conducted to comprehensively test the capability and applicable situations of COM graph, TIN graph and classical RANSAC (Section 3). Finally, this paper is summarized (Section 4).

## 2. Methods

The height variations of terrain and building cause some geometric deformation in the stereo pair of remote sensing images. As the ratio of the height variations to altitudes of the image sensors is very small, the geometric deformation is generally in a small range, that is, the local geometric features of the image pairs are almost invariant and similar. The complete graph can provide sufficient measurements of local geometric similarity to estimate meaningful statistical parameters, which can be used to distinguish outliers from inliers. Each node is connected with other nodes in the complete graph. When there are adequate matching points, TIN graph can effectively retain some geometric properties of the complete graph and hugely reduce computational complexity. Thus, we use TIN graph to approximate the complete graph and propose a TIN graph-based method. In the following section, we will provide a detailed descriptions of the complete graph-based method and the TIN graph-based method for matching blunder detection.

### 2.1. Complete Graph-Based Method (COM Graph)

**Figure 1:**
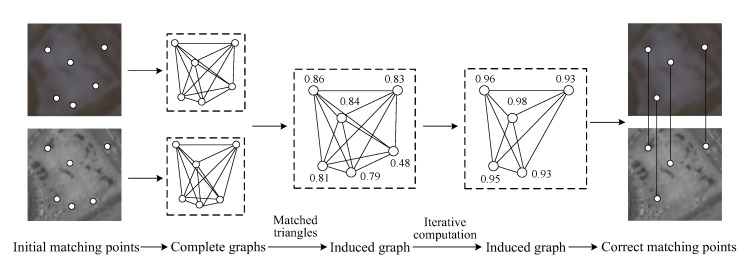
The workflow of COM graph.

Given two initial matching point sets in the image pair from different sensors, the complete graph-based method (COM graph) aims to detect the matching blunders between the two matching point sets. COM graph involves three steps (shown in Figure 1). (1) Building the complete graphs. Two complete graphs are built with the two matching point sets, respectively. (2) Building the induced graph. The induced graph is computed via similarities of all matched triangle pairs between the two complete graphs. (3) Iterating the induced graph. Matching blunders can be detected by iteratively deleting the node of the minimum attribute value and rebuilding the induced graph until the minimum attribute value meets the given condition.

A complete graph is a simple undirected graph in which every pair of distinct nodes is connected by a unique edge, and nodes of the complete graph represent corresponding matching points. As shown in Figure 1 in the second column, there are 20($C_{6}^{3}$) matched triangle pairs between the two complete graphs, and for each node pair, there are 10 matched triangle pairs that contain this node pair. That is, the number of matched triangle pairs is much larger than the number of nodes in complete graphs. Therefore, complete graphs can construct statistically significant clusters of matched triangle pairs to encode local geometric similarity for detecting matching blunders. However, too many matched triangles between complete graphs result in huge computational complexity of COM graph. Thus, we use TIN graph to approximate the complete graph to improve computational efficiency and, for this reason, we propose the TIN graph-based method.

### 2.2. TIN Graph-Based Method (TIN Graph)

**Figure 2:**
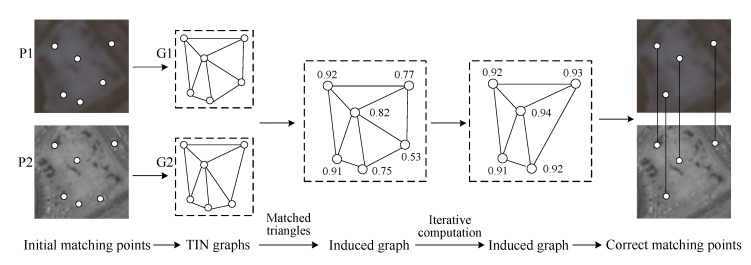
The workflow of triangular irregular network (TIN) graph.

Suppose there are *n* pairs of matching points in the image pair, the number of matched triangle pairs in the two complete graphs is approximately proportional to *n*^3^, and this leads to redundant information and high computational complexity of COM graph when the number *n* is very large. In contrast, the number of matched triangle pairs in two TIN graphs is approximately proportional to *n*, and TIN graph can effectively retain some geometric properties of the complete graph in the case of abundant matching points and relatively low matching blunder ratios. Thus, complete graphs can be approximated by TIN graphs for improving computational efficiency in some applicable situations. The proposed TIN graph-based method (TIN graph, shown in Figure 2) also contains three steps: (1) Building the TIN graphs. (2) Building the induced graph. (3) Iterating the induced graph. The detailed descriptions of the three steps are as follows:

#### 2.2.1. Building the TIN Graphs

A TIN graph is a simple undirected graph which is composed of Delaunay triangles, and nodes of the TIN graph represent corresponding matching points. As shown in Figure 2 in the first and second column, both matching point sets P1 and P2 contain six matching points in the image pair, and two TIN graphs G1 and G2 are built with sets P1 and P2 to encode local geometric similarity for matching blunder detection, respectively. Furthermore, there are five matched triangle pairs between the two TIN graphs, while the number of matched triangle pairs between the two complete graphs is 20 (shown in Figure 1 in the second column). Thus, in the case of the same number of matching point pairs, TIN graph has a much smaller number of matched triangle pairs than COM graph.

#### 2.2.2. Building the Induced Graph

**Figure 3:**
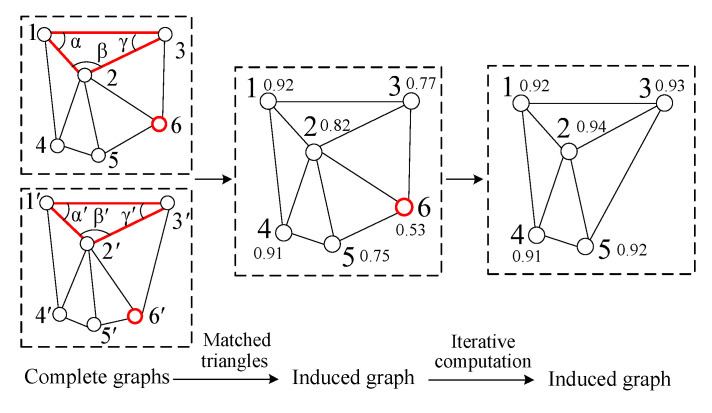
The main workflow of building and iterating the induced graph.

The induced graph is the TIN graph with node attribute values that indicate the local geometric similarity of the matching points (shown in Figure 3). There are two steps for building the induced graph: (1) Calculate the similarity of matched triangles. (2) Calculate the attribute value of the induced graph node. The detailed descriptions of the two steps are as follows:

(1) Calculating the similarity of matched triangles

As shown in Figure 3 in the first column, $\left(1,1^{\prime }\right)$ $\left(2,2^{\prime }\right)$ $\left(3,3^{\prime }\right)$ are three pairs of corresponding nodes in the two complete graphs, and triangles Δ123 and $\Delta 1^{\prime }2^{\prime }3^{\prime }$ are two corresponding matched triangles. The similarity between the two triangles can be computed by Equation (1) [16,24]:

(1) $$s(t)=exp[-\frac{1}{\varepsilon^{2}}{\left(\left\|t_{\Delta 123}-t_{\Delta 1^{\prime }2^{\prime }3^{\prime }}\right\|\right)}^{2}]$$

where s(t) is the similarity between triangles Δ123 and $\Delta 1^{\prime }2^{\prime }3^{\prime }$, ε is the Gaussian kernel band width (its value is 1 in this paper), ${\left\|.\right\|}_{2}$ is the length of a vector, the vectors $t_{\Delta 123}$ and $t_{\Delta 1^{\prime }2^{\prime }3^{\prime }}$ are the geometric descriptors of triangles Δ123 and $\Delta 1^{\prime }2^{\prime }3^{\prime }$.

The geometric descriptor of a triangle is usually expressed as cosines of three interior angles in the triangle, and they are invariant to translation, rotation and scaling. The triangle descriptors of triangles Δ123 and $\Delta 1^{\prime }2^{\prime }3^{\prime }$ can be computed by Equation (2):

(2) $$t_{\Delta 123}=(\cos\alpha ,\cos\beta ,\cos\gamma ),t_{\Delta 1^{\prime }2^{\prime }3^{\prime }}=(\cos\alpha^{\prime },\cos\beta^{\prime },\cos\gamma^{\prime })$$

where vectors $t_{\Delta 123}$ and $t_{\Delta 1^{\prime }2^{\prime }3^{\prime }}$ are the geometric descriptors of triangles Δ123 and $\Delta 1^{\prime }2^{\prime }3^{\prime }$, (α,β,γ) and $\left(\alpha^{\prime },\beta^{\prime },\gamma^{\prime }\right)$ are the interior angles of triangles Δ123 and $\Delta 1^{\prime }2^{\prime }3^{\prime }$, respectively (shown in Figure 3 in the first column).

(2) Calculating the similarity of matched triangles

The attribute value of one node is the mean value of summing up similarities of matched triangle pairs that contain this node. The number of all nodes in the induced graph is set to *n*, the number of matched triangle pairs that contain the node *i* (1≤i≤n) is $N_{i}$ in TIN graphs. Then these $N_{i}$ matched triangle pairs can be constructed as one cluster *I* = {$\left(\Delta ijk;\Delta i^{\prime }j^{\prime }k^{\prime }\right)$, $\left(\Delta imn;\Delta i^{\prime }m^{\prime }n^{\prime }\right)$, $\left(\Delta ipq;\Delta i^{\prime }p^{\prime }q^{\prime }\right)$, …}. Each element in the cluster *I* represents one matched triangle pair *I_t_*, the cluster *I* can be expressed as:

(3) $$I=\{1\leq t\leq N_{i},t\in IR|I_{t}\}$$

Then, the attribute value $v_{i}$ of the node *i* can be calculated by Equation (4):

(4) $$v_{i}=\frac{1}{N_{i}}\sum_{t=1}^{t=N_{i}}s(I_{t})$$

where $v_{i}$ is the attribute value of the node *i* in the induced graph, s(t) is the similarity of one triangle pair $I_{t}$, and $N_{i}$ is the number of all triangle pairs in the cluster *I*.

The computing method of the node attribute $v_{i}$ can be observed in Figure 3 in the first column. The cluster *I* of node 2 has five triangle pairs, namely $\left\{(\Delta 123;\Delta 1^{\prime }2^{\prime }3^{\prime }\right)$, $\left\{(\Delta 124;\Delta 1^{\prime }2^{\prime }4^{\prime }\right)$, $\left\{(\Delta 245;\Delta 2^{\prime }4^{\prime }5^{\prime }\right)$, $\left\{(\Delta 256;\Delta 2^{\prime }5^{\prime }6^{\prime }\right)$, and $\left\{(\Delta 236;\Delta 2^{\prime }3^{\prime }6^{\prime }\right)$, the similarities of the above-mentioned triangle pairs can be computed by Equation (1), and the attribute value of node 1 can be calculated by Equation (4). Then, we can obtain the attribute values of all nodes and build the induced graph (shown in Figure 3 in the second column).

#### 2.2.3. Iterating the Induced Graph

The matched triangle pairs in the cluster *I* of node *i* can be divided into the two following cases: (1) matched triangle set $I_{a}$—all matched triangles consist of correct matching points, and (2) matched triangle set $I_{b}$—every matched triangle in the set has at least one erroneous matched point. Therefore, one triangle pair in $I_{a}$ has larger similarity than one triangle pair in $I_{b}$.

Suppose there are $\#(I_{a})$ and $\#(I_{b})$ pairs of matched triangles in $I_{a}$ and $I_{b}$, respectively; and the sum of similarities of matched triangles in $I_{a}$ and $I_{b}$ are $\sum_{t=1}^{t=\#(I_{a})}\left[s(I_{t})\right]$ and $\sum_{t=1}^{t=\#(I_{b})}\left[s(I_{t})\right]$, respectively. Thus, the attribute value $v_{i}$ of node *i,* which contains the cluster $I_{a}$ and $I_{b}$, is $v_{i}=\frac{1}{\#(I_{a})+\#(I_{b})}\{\sum_{t=1}^{t=\#(I_{a})}\left[s(I_{t})\right]+\sum_{t=1}^{t=\#(I_{b})}\left[s(I_{t})\right]\}$. Then, Equation (4) can be transformed as:

(5) $$v_{i}=[s(I_{a})+s(I_{b})]/[\#(I_{a})+\#(I_{b})]$$

where *v_i_* is the attribute value of node *i* in the induced graph, $s(I_{a})$ is the sum of similarities of matched triangles in $I_{a}$, $\#(I_{a})$ is the number of matched triangles in $I_{a}$, as are $s(I_{b})$ and $\#(I_{b})$. The nodes in the induced graph represent matching points in the image pair. A correct matching point has the cluster that contains $I_{a}$ and $I_{b}$, and a matching blunder has the cluster that only contains $I_{b}$. Thus, a correct matching point generally has a larger attribute value than a matching blunder, and the attribute value of a node represents the probability that the node is a matching blunder, namely, matching blunder detection can be realized by eliminating the node with a smaller attribute value. For example, as shown in Figure 3 in the second and third column, node 6 has a much smaller attribute value than the other nodes (1 to 5), so it is a matching blunder and should be eliminated.

However, in the case of high matching blunder ratios, the cluster *I* of a correct matching point contains small $I_{a}$ and large $I_{b}$, that is, a ratio of $\#(I_{a})$ to $\#(I_{b})$ may be very low, and the ratio is roughly inversely proportional to the matching blunder ratio. Thus, attribute values of some correct matching points may be very close to those of matching blunders, and correct matching points are likely to be treated as matching blunders. In order to avoid this case, we adopt the iterative algorithm to filter induced graph nodes that represent matching blunders (algorithm details are illustrated in Algorithm 1).

**Algorithm 1**: Matching blunder detection by iterating the induced graph

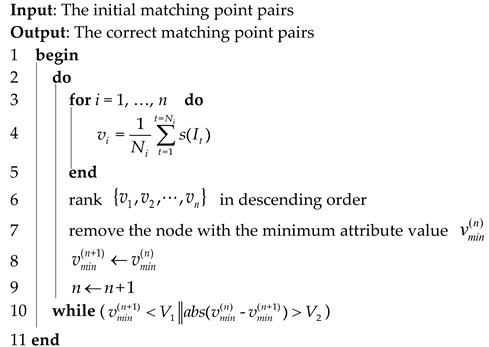



In Algorithm 1, the threshold $V_{1}$ for TIN graph can be calculated by Equation (4) based on the correct matching points in the TIN graphs, and the threshold $V_{2}$ is generally 0.01. This is also the case for the thresholds $V_{1}$ and $V_{2}$ in COM graph. When there are too many matching points in the image pair, the iterative computation of Algorithm 1 for COM graph will be too complex, leading to excessive computation time. Therefore, the image pair can be divided into small regions first, and then Algorithm 1 can be used to detect matching blunders. Contrastingly, TIN graph generally can achieve much higher computational efficiency even applying Algorithm 1 to the whole image pair.

### 2.3. Differences between TIN Graph and COM Graph

When comparing Figure 1 and Figure 2, it is clear that there are much fewer matched triangle pairs in TIN graphs than in complete graphs, which results in different performances of computational complexity and robustness for matching blunder detection. The reasons are as follows:

Firstly, fewer matched triangle pairs of TIN graph result in higher computational efficiency than COM graph. Suppose there are *n* pairs of matching points in the image pair, the number of matched triangle pairs in two complete graphs and two TIN graphs is approximately proportional to *n*^3^ and *n*, respectively. As the main iterative calculation steps (step 3 to step 9 of Algorithm 1) for COM graph and TIN graph are all based on matched triangle pairs, TIN graph can greatly reduce the overall computational complexity.

**Figure 4:**
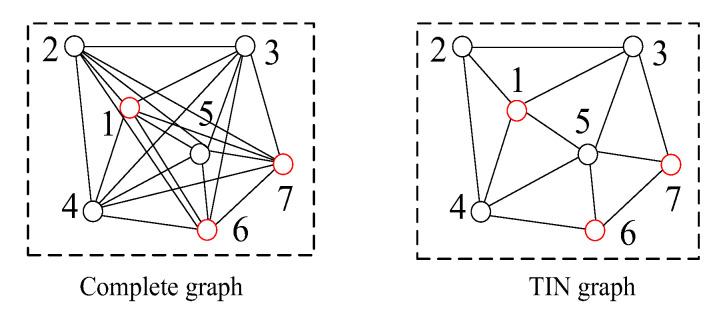
Comparison of the complete graph and TIN graph.

Secondly, fewer matched triangle pairs of TIN graph lead to lower robustness than COM graph. In the case of high matching blunder ratios, a correct matching point may be surrounded by matching blunders in graphs. For example, as shown in Figure 4, nodes 1, 6, 7 represent correct matching points, and nodes 2 to 5 represent matching blunders. Node 1 is affected by nodes 2 to 5 in TIN graph and has a small cluster that only contains $I_{b}$, its attribute value is too small to be distinguished from matching blunders and even Algorithm 1 cannot work effectively. Contrarily, node 1 is affected by nodes 1 to 7 in the complete graph, thus COM graph can ensure that a correct matching point has a larger cluster which contains a certain number of both $I_{a}$ and $I_{b}$, and its attribute value is generally larger than a matching blunder.

Thus, only in the case of abundant matching points and relatively low matching blunder ratios should complete graphs be simplified by TIN graphs. In Section 3, we will use simulated data experiments to show within what range of matching blunder ratios COM graph can be replaced by TIN graph.

Besides, compared with complete graphs, all matched triangles in TIN graphs are in a small local image, thus, their geometric features are barely affected by image deformation. Therefore, matched triangles in $I_{a}$ for TIN graph have higher similarities than most of matched triangles in $I_{a}$ for COM graph, such as Δ123 and Δ267 in Figure 4 (nodes 1, 2, 3, 6, 7 are assumed to be correct matching points here). In the case of relatively low matching blunder ratios, ratios of $\#(I_{a})$ to $\#(I_{b})$ for TIN graph and COM graph are approximately equal and relatively high. According to Equation (5), TIN graph can generally obtain a larger attribute value for the same correct matching point than COM graph, and is less likely to regard correct matching points as matching blunders. Capabilities and applicable situations of COM graph and TIN graph will be discussed in experiments of simulated data and real data in Section 3. Generally, TIN graph are less robust in matching blunder detection, and have much less computational complexity and high detection efficiency.

## 3. Experimental Results and Discussions

In this section, we select four pairs of complete images captured by the sensors of Gaofen1 (GF1), near infrared ray of Gaofen1 (GF1-NIR), Gaofen2 (GF2), panchromatic Landsat (Landsat-PAN), Ziyuan3 (ZY3), Jilin1 (JL1) and digital camera of unmanned aerial vehicle (UAV) as real dataset, and extract four pairs of sub-images from the above complete images as a simulated dataset. Then, based on six evaluation criteria, experiments of simulated data and real data are conducted to comprehensively test and compare the capability and applicable situations of COM graph, TIN graph and classical RANSAC. The simulated data experiments and real data experiments are presented in detail in the following sections.

### 3.1. Simulated Data Experiments

**Figure 5:**
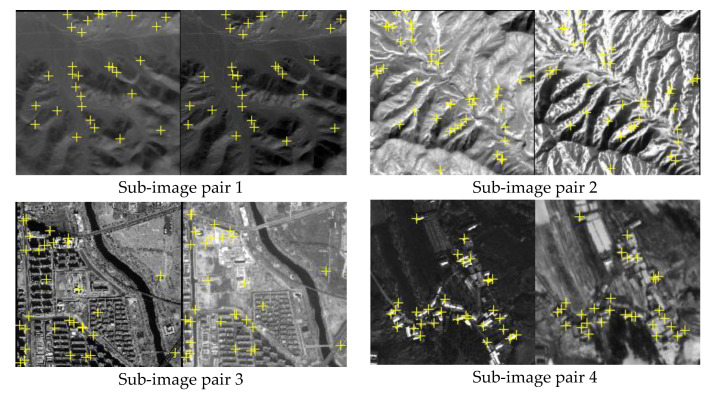
Manually measured tie points (yellow crosses) in four sub-image pairs.

The simulated dataset includes four sub-image pairs with 400 × 400 pixels. As shown in Figure 5, sub-image pairs 1 to 4 are from GF1-NIR and Landsat-PAN, GF-1 and ZY-3, GF-2 and ZY-3, and JL-1 and UAV, respectively. Moreover, 30, 40, 30 and 30 evenly distributed correct matched points were manually selected as the ground truth in the four sub-image pairs, respectively. The ground truth represents the confirmed correct matching points between image pairs, used as benchmarks to evaluate the performance of detection methods in the experiments. In order to test the capability of the methods for different matching blunder ratios, seven sets of evenly distributed matching blunders are randomly generated and added into the above four sets of correct tie points, and the matching blunder ratios are set from 10% to 70%. All the matching blunders are also manually confirmed.

In order to evaluate the proposed methods in the simulated data experiments, we use three criteria including recognition rate *a*, false rate *b*, and computation time *t*. The practically used detection method is likely to detect most, but not all, matching blunders from matching points in practice, and may falsely treat correct matching points as matching blunders. That is to say, detection methods have different effects of (1) recognizing matching blunders correctly and (2) misjudging correct matching points. These two effects can be quantified by two criteria: (1) recognition rate *a*, (2) false rate *b*. Besides, recognition rate *a* is equal to the recall rate (i.e., the ratio of detected matching blunders to the total matching blunders).

Suppose the initial matching point set contains *f* pairs of matching blunders and *c* pairs of correct matching points. After processing by a detection method, we can obtain a detection result of matching blunders. Suppose the detection result contains $df_{1}$ pairs of true matching blunders and $dc_{1}$ pairs of correct matching points. Then, recognition rate *a* and false rate *b* can be calculated as follows:

(6) $$a=\frac{df_{1}}{f},b=\frac{dc_{1}}{c}$$

Recognition rate *a* measures the positive effect of the matching blunder detection method, and false rate *b* measures the negative effect, that is, greater *a* and smaller *b* are always favorable in experiments and real tasks.

**Figure 6:**
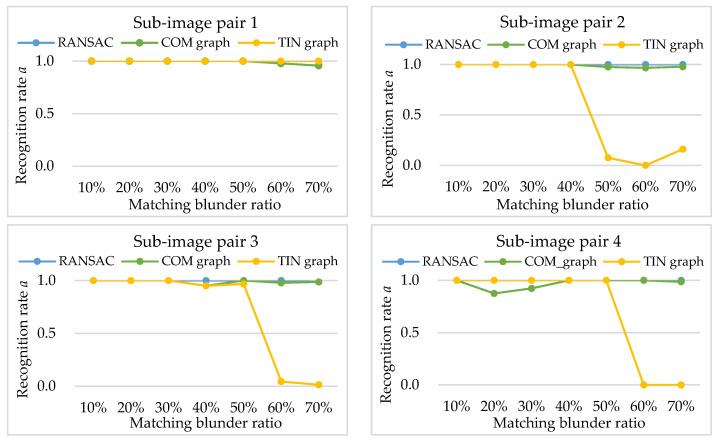
Experimental results of recognition rate *a*.

**Figure 7:**
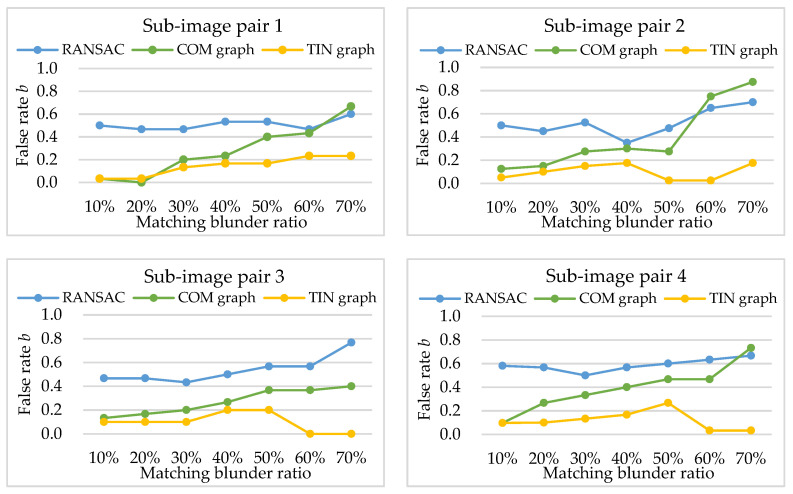
Experimental results of false rate *b*.

Then, RANSAC, COM graph and TIN graph are conducted in the simulated data, and the experimental results of recognition rate *a*, false rate *b*, and computation time *t* are shown in Figure 6, Figure 7 and Table 1, respectively. In the RANSAC scheme, the homography transformation models are embedded, as the model can give good approximation to the geometric relationship of pine hole and push-broom sensors [26]. Furthermore, the back-projective error threshold of the homography transformation model is set to 3.0 pixels.

The experimental results of recognition rate *a* (shown in Figure 6) indicate different positive effect of matching blunder detection for the three methods. With an increase in matching blunder ratios, the recognition rates of RANSAC and COM graph are mostly around 1.0, indicating that the two methods can detect nearly all the matching blunders. However, the recognition rates of TIN graph decrease rapidly when the matching blunder ratios exceed 50% (shown in Figure 6 sub-image pairs 2, 3, 4). As described in Section 2.3, when the matching blunder ratio is too high, a correct matching point is mostly surrounded by matching blunders, and has a small cluster that only contains $I_{b}$ in TIN graphs. Therefore, the attribute value of the correct matching point calculated by TIN graph nearly equals a matching blunder, that is, TIN graph cannot effectively detect matching blunders. On the other hand, a correct matching point has a very large cluster that contains a number of $I_{a}$ and $I_{b}$ in complete graphs, and its attribute value is generally larger than a matching blunder. Thus, COM graph can ensure high recognition rates in spite of high matching blunder ratios, and is more robust than TIN graph. Nevertheless, if matching blunder ratio is lower than 50%, COM graph can be replaced by TIN graph for higher computational efficiency. Alternatively, Algorithm 1 can be transform to a hybrid of TIN graph and COM graph.

Experimental results of false rate *b* (shown in Figure 7) indicate that RANSAC has worse negative effect of matching blunder detection than COM graph and TIN graph, namely, it falsely treats the largest number of correct matching points as matching blunders. The mean false rates of RANSAC and COM graph in the four sub-image pairs are 0.3 and 0.5, respectively. Meanwhile, the false rates of TIN graph are mostly no more than 0.2 within relatively low matching blunder ratios (lower than 50%). The reason is that RANSAC randomly samples matching points to fit the global approximate transformation model (homography transformation). Both high matching blunder ratios and large local deformation in the four sub-image pairs lead to large errors in the homography transformation model and the tendency to trap in local optimum; thus, a large number of correct matching points are falsely treated as matching blunders. However, both COM graph and TIN graph are local similarity-based methods that adopt strategies of clustering matched triangles and iterating induced graphs. Therefore, their false rates are less affected by local image deformation and matching blunder ratios, and are generally lower than RANSAC.

Within a relatively low matching blunder ratio (generally no higher than 50%), TIN graph has smaller false rates than COM graph. This can be explained as follows: Let $I_{a\_\mathrm{TIN}}$ and $I_{b\_\mathrm{TIN}}$ be the matched triangle sets $I_{a}$ and $I_{b}$ for TIN graph, and $I_{a\_\mathrm{COM}}$ and $I_{b\_\mathrm{COM}}$ be the matched triangle sets $I_{a}$ and $I_{b}$ for COM graph. Apparently, $I_{a\_\mathrm{COM}}$ contains $I_{a\_\mathrm{TIN}}$. As analyzed in Section 2.3, matched triangles in $\left(I_{a\_\mathrm{COM}}-I_{a\_\mathrm{TIN}}\right)$ cover a larger area of the sub-images than matched triangles in $I_{a\_\mathrm{TIN}}$, thus matched triangles in $\left(I_{a\_\mathrm{COM}}-I_{a\_\mathrm{TIN}}\right)$ are affected by larger image deformation and have lower similarities. Besides, within the relatively low matching blunder ratios, the ratio of $\#(I_{a\_\mathrm{COM}})$ to $\#(I_{b\_\mathrm{COM}})$ is approximately equal to the ratio of $\#(I_{a\_\mathrm{TIN}})$ to $\#(I_{b\_\mathrm{TIN}})$. According to Equation (5), TIN graph can generally obtain a larger attribute value for the same correct matching point than COM graph, namely, TIN graph is less likely to treat the correct matching point as a matching blunder.

In the experiments of four pairs of sub-images, there are two remarkable characteristics of computation time for the three methods (shown in Table 1). (1) The computation time of the three methods presents an increasing trend with an increase in matching blunder ratios. This is because RANSAC needs more iterations to generate a correct sample, and both COM graph and TIN graph need more time to iterate induced graphs (illustrated in Algorithm 1). (2) The computational complexity of TIN graph nearly equals that of RANSAC, while COM graph has a much higher computational complexity than TIN graph and RANSAC. The reason is that COM graph needs plenty of time to build complete graphs and induced graphs in the same situation (as analyzed in Section 2.3).

### 3.2. Real Data Experiments

**Figure 8:**
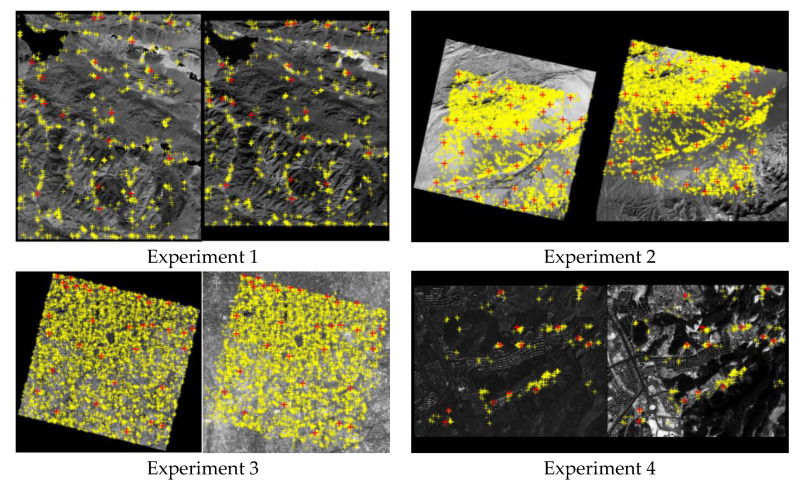
All the obtained matching points in the four complete image pairs. Yellow crosses represent the initial matching points and red crosses represent the checkpoints.

The main parameters of the four image pairs in a real dataset are shown in Table 2, all image pairs are typically characterized with non-linear density differences and considerable textural changes. As shown in Figure 8, experiments 1 to 4 represent the four complete images pairs of GF1-NIR and Landsat-PAN, GF-1 and ZY-3, GF-2 and ZY-3, and JL-1 and UAV, respectively. We adopt the matching algorithm based on graph theory [27] to obtain 898, 10,523, 7178, and 231 pairs of initial matching points, and manually measure evenly distributed 22, 41, 34 and 13 pairs of correct matching points as the checkpoints, respectively. These checkpoints are the ground truth in the performance evaluation of the proposed algorithms.

In order to evaluate the proposed methods in the real data experiments, we use four criteria, namely, number of remaining matching point pairs, dispersion, positional accuracy (RMSE—root mean square error), and computation time. Dispersion and positional accuracy are determined as follows: Dispersion represents the spatial distribution of the matching points in the image, and it is closely related to the positional accuracy of satellite images. Dispersion of the matching points can be computed by Equation (7) [28]:

(7) $$D=D_{S}\times D_{A}=\sqrt{\sum_{i=1}^{n}{\left(A_{i}/\bar{A}-1\right)}^{2}/\left(n-1\right)}\times \sqrt{\sum_{i=1}^{n}{\left(S_{i}-1\right)}^{2}/\left(n-1\right)}$$

where *D* is the dispersion of the matching points, *n* is the number of all triangles in the TIN constructed by matching points, $D_{S}$ is the quantified value of area changes of all triangles, $D_{A}$ is the quantified value of shape changes of all triangles, $A_{i}$ is the area of triangle *i*, $\bar{A}$ is the average area of all triangles, $S_{i}$ is the largest interior angle of triangle *i*. Smaller dispersion of the matching points means more uniform spatial distribution of the matching points. The larger dispersion value between an image pair is generally selected as the final dispersion *D*.

The positional accuracy (RMSE) of satellite images is usually calculated by the method of TIN analysis [29]:

(8) $$\mathrm{RMSE}=\sqrt{\sum_{i=1}^{n}{\left(X_{i}-X_{i}^{\prime }\right)}^{2}/n}$$

where *n* is the number of checkpoints, $X_{i}$ is the real coordinates of checkpoint *I* in image plane, and $X_{i}^{\prime }$ is the coordinates of checkpoint *i* calculated by the corresponding affine transformation model. First, two TINs are constructed using all matching points of an image pair, and *n* pairs of evenly distributed checkpoints are added to the Delaunay triangle pairs in the two TINs. Then, an affine transformation model is fitted by the triangle pairs with checkpoint *i*, and $X_{i}^{\prime }$ can be calculated. Lastly, RMSE is calculated through *n* pairs of checkpoints.

**Figure 9:**
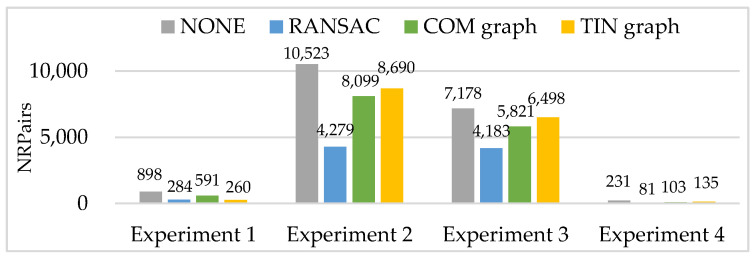
Experimental results of the number of remaining matching point pairs (NRPairs).

**Figure 10:**
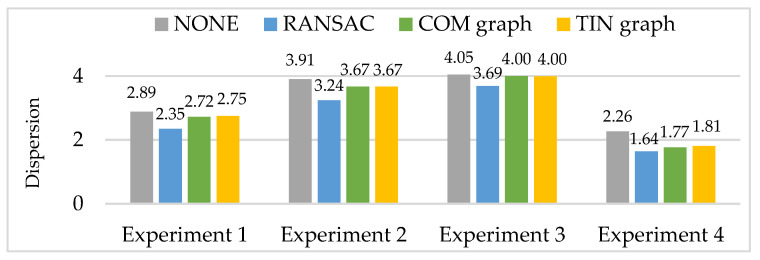
Experimental results of dispersion *D*.

**Figure 11:**
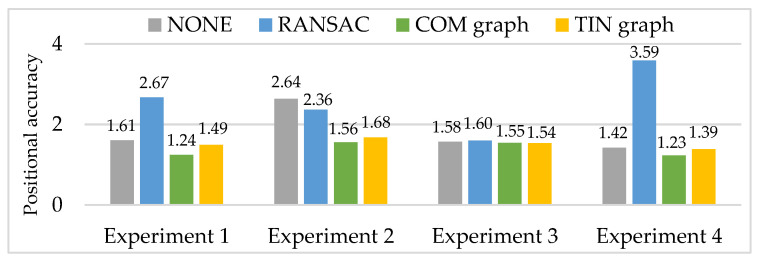
Experimental results of positional accuracy (RMSE, pixel).

Then, RANSAC, COM graph and TIN graph are conducted in the real data, and experimental results of a number of remaining matching point pairs (NRPairs), dispersion, positional accuracy, and computation time are shown in Figure 9, Figure 10 and Figure 11 and Table 3, respectively. Additionally, NONE represents the statistical results of the initial matching points which are not handled with any detection methods.

The number of remaining matching point pairs (NRPairs) is closely related to the recognition rate and false rate of the matching blunder detection method. As shown in Figure 9, RANSAC acquires much less NRPairs than COM graph and TIN graph in experiments 1 to 4, and TIN graph acquires more NRPairs than COM graph in experiments 2 to 4. The experimental results of NRPairs indicate that TIN graph can work effectively and matching blunder ratios of the four experiments are within relatively low ranges (no higher than 50%). Thus, the three methods have a recognition rate of about 1.0 and can almost detect all matching blunders in experiments 1 to 4. Besides, a higher false rate means the method falsely treats more correct matching points as matching blunders. Thus, the NRPairs of RANSAC is slightly less than that of COM graph, and much less than that of TIN graph in experiments 2 to 4.

However, TIN graph acquires the least NRPairs in experiment 1. The reason is that high mountains in the whole images of experiment 1 lead to huge local image deformation, and the matching triangles in $I_{a}$ for TIN graph and COM graph are all affected by large image deformation and have relatively low similarities. Moreover, the matching points distribute relatively sparsely and not evenly, that is, a correct matching point may be surrounded by matching blunders. Therefore, TIN graph is more likely to falsely treat a correct matching point as a matching blunder and obtain a very high false rate in experiment 1, while COM graph has a much larger matched triangle set $I_{a}$, and is more successful at maintaining its low false rate.

In order to easily show and compare the experimental results of dispersion, we first take the base-10 logarithm of the initial dispersion, then take the negative of the previous results to obtain the dispersion *D* in the four experiments (shown in Figure 10). Thus, larger dispersion *D* in Figure 10 means more even spatial distribution of the matching points. In experiments 1 to 4, all the three methods have smaller dispersion *D* than NONE, indicating that spatial distribution of the matching points becomes worse. Meanwhile, RANSAC has the smallest dispersion *D*, namely, the worst spatial distribution of the matching points. Additionally, COM graph and TIN graph have nearly equal dispersion *D*.

Larger NRPairs, better dispersion and a lower matching blunder ratio generally mean better positional accuracy of the remaining matching points. Since all the experimental results of the three methods have low and approximately equal matching blunder ratios, and according to the experimental results of NRPairs (shown in Figure 9) and dispersion (shown in Figure 10), RANSAC should have a much worse positional accuracy than COM graph and TIN graph (shown in Figure 11). Compared with NONE, the positional accuracy of RANSAC is somewhat better in experiment 2, is slightly worse in experiment 3, and is especially worse in experiment 1 and experiment 4. Meanwhile, both COM graph and TIN graph obtain a better positional accuracy than NONE in experiments 1 to 4. Moreover, COM graph has overall a higher positional accuracy than TIN graph. Though RANSAC, COM graph and TIN graph all have smaller NRPairs, worse dispersion and lower matching blunder ratios than NONE, their positional accuracies have remarkably different performances. The reason is that COM graph and TIN graph have much larger NRPairs and better dispersion than RANSAC, and this may be somehow related to the calculation method of positional accuracy (Equation (8)).

Computation time results of the three methods (shown in Table 3) indicate two significant characteristics in real data experiments. (1) TIN graph has the almost equal computational complexity to RANAC, and they have much lower computational complexity than COM graph, as verified in the simulated data experiments (shown in Table 1). (2) The computation time of RANSAC is related to image terrain, while both COM graph and TIN graph are barely affected by the terrain. When geometric deformation between images is large, traditional homography transformation adopted by RANSAC cannot express the geometric relationship between images accurately, thus, it needs more iterations to produce an optimal consensus. Contrarily, both COM graph and TIN graph adopt clusters of matched triangle pairs to encode the local similarity of images, and they are independent of geometric deformation between images.

## 4. Conclusions

To solve the matching blunder detection problems of multi-source remote sensing images, namely, fewer matching points with higher matching blunder ratios and unknown global geometric relationship models between images, we studied the complete graph-based method (COM graph) and the TIN graph-based method (TIN graph). Both the above-mentioned methods first construct clusters of matched triangle pairs in two graphs to encode the local geometric similarity of images, then calculate node attribute values of the induced graph to identify matching blunders.

Based on simulated data and real data experiments, both COM graph and TIN graph can acquire reliable detection results and outperform the classical RANSAC method in terms of recognition rate, false rate, the number of remaining matching point pairs, dispersion, and positional accuracy in most cases. This is because the biggest disadvantage of RANSAC is that it cannot handle large local geometric deformation, especially in multi-source image pairs. In contrast, both COM graph and TIN graph are rarely affected by geometric deformation.

In terms of multi-source images with large local geometric deformation, (1) in the case of high matching blunder ratios (10% to 70%), COM graph can achieve a better performance of detecting matching blunders than RANSAC, and it has a higher computational complexity and requires much more computation time; (2) in the case of relatively low matching blunder ratios (generally no higher than 50%), TIN graph can obtain better detection results than RANSAC, and the computation time of TIN graph and RANSAC is approximately equal.

However, the proposed methods can only detect matching blunders, and cannot give an optimal geometric model between multi-source images when the factor of matching noise is considered. Furthermore, the proposed TIN graph has the disadvantage that it will not work if the matching blunder ratio is higher than 50%. Besides, in experiments of simulated data and real data, the two graph theory-based methods, namely COM graph and TIN graph, can only handle one pair of multi-source images at a time. It is worth considering how to deal with matching blunders of multi-view images via graph theory, as well as further enhancing the robustness of the proposed methods.

## Figures and Tables

**Table 1:** Experimental results of computation time *t* (ms, *t* within 1 ms is not recorded). RANSAC—classical random sample consensus.

	Matching Blunder Ratio	10%	20%	30%	40%	50%	60%	70%
Sub-image pair 1	RANSAC	<1	<1	15	16	47	47	47
	COM graph	<1	<1	16	31	63	110	250
	TIN graph	<1	16	<1	15	15	31	62
Sub-image pair 2	RANSAC	<1	<1	<1	<1	31	47	46
	COM graph	15	31	47	62	125	266	532
	TIN graph	<1	16	16	31	<1	<1	62
Sub-image pair 3	RANSAC	<1	16	<1	15	47	62	47
	COM graph	15	31	32	47	78	140	296
	TIN graph	<1	<1	15	16	16	<1	<1
Sub-image pair 4	RANSAC	16	15	15	32	47	47	46
	COM graph	<1	16	31	46	63	109	250
	TIN graph	<1	<1	<1	<1	15	<1	16

**Table 2:** Parameters of experimental complete image pairs.

No.	Image Pair	Landform	Image Size (Pixels)	GSD (m)	Acquisition Date
1	GF1-NIR	High mountain	5108 × 6255	2.0	November 2013
	Landsat-PAN		5438 × 6090	2.1	July 2013
2	GF-1	Mountain	20,766 × 20,662	2.0	November 2013
	ZY-3		30,008 × 30,100	2.1	December 2012
3	GF-2	Urban area	9375 × 9231	1.0	February 2015
	ZY-3		20,516 × 20,613	2.1	October 2012
4	JL-1	Hill	4765 × 4070	1.0	July 2014
	UAV		4990 × 3908	0.5	January 2014

**Table 3:** Experimental results of computation time *t* (ms).

	Experiment 1	Experiment 2	Experiment 3	Experiment 4
NONE	-	-	-	-
RANSAC	17	27	8	12
COM graph	52	1034	691	10
TIN graph	13	31	16	2
